# Exploiting the molecular subtypes and genetic landscape in pancreatic cancer: the quest to find effective drugs

**DOI:** 10.3389/fgene.2023.1170571

**Published:** 2023-09-18

**Authors:** Nnenna Elebo, Ebtesam A. Abdel-Shafy, Stefano Cacciatore, Ekene Emmanuel Nweke

**Affiliations:** ^1^ Department of Surgery, Faculty of Health Sciences, University of Witwatersrand, Johannesburg, Gauteng, South Africa; ^2^ Bioinformatics Unit, International Centre for Genetic Engineering and Biotechnology, Cape Town, South Africa; ^3^ National Research Centre, Cairo, Egypt

**Keywords:** Pancreatic Cancer, drug repurposing, mutations, genomics, precision medicine

## Abstract

Pancreatic Ductal Adenocarcinoma (PDAC) is a very lethal disease that typically presents at an advanced stage and is non-compliant with most treatments. Recent technologies have helped delineate associated molecular subtypes and genetic variations yielding important insights into the pathophysiology of this disease and having implications for the identification of new therapeutic targets. Drug repurposing has been evaluated as a new paradigm in oncology to accelerate the application of approved or failed target-specific molecules for the treatment of cancer patients. This review focuses on the impact of molecular subtypes on key genomic alterations in PDAC, and the progress made thus far. Importantly, these alterations are discussed in light of the potential role of drug repurposing in PDAC.

## 1 Introduction

Pancreatic Ductal Adenocarcinoma (PDAC) is the most common form of pancreatic cancer accounting for more than 90% of all pancreatic malignancies (Kleeff et al., 2016). This aggressive cancer, with a 5-year survival rate of about 12%, is one of the leading causes of cancer-related deaths worldwide (Siegel et al., 2022). It is predicted to be the second most common cause of cancer-related death in the United States by 2030 (Rahib et al., 2014). In low-to-middle-income countries such as South Africa, it is currently the seventh leading cause of cancer-related death (Statistics South Africa, 2023). Surgery remains the best treatment strategy. In recent years, there have been significant technological advancements which have led to elucidating key molecular mechanisms involved in the progression of PDAC. Several omics analyses such as genomics, transcriptomics, and metabolomics have been used to demonstrate the heterogeneity of PDAC between different tumours (inter-tumour) and within the same tumour (intra-tumour) (Cros et al., 2018; Elebo et al., 2021; Gutiérrez et al., 2021). The genetic landscape of pancreatic cancer explores the intricate genetic alterations that drive the initiation, progression and metastasis of pancreatic cancer. Examples include mutations in key genes such as *KRAS*, *SMAD4*, *P53*, and *CDKN2A* observed in pancreatic cancer (Falasca et al., 2016). In this era of precision medicine, these identified mechanisms can be exploited for diagnosis, treatment and management (Halbrooks et al, 2023). These emerging therapeutic approaches offer a glimpse into the discovery and development of novel effective drugs that could emerge from our improved understanding of the molecular subtypes and genetic landscape (Elebo et al., 2020; Nsingwane et al., 2020; Wang et al., 2021a). However, unlike other solid tumours, pancreatic cancer continues to show resistance to some of these novel therapies. This characteristic together with the lengthy and expensive process of the development of new drugs impedes the closing of the gap observed between our intricate understanding of the molecular and genetic landscape of PDAC and clinical benefit to patients. To leverage this understanding, consideration should be given to repurposing other well-known drugs for the treatment of the disease.

Hence this mini-review delves into the current knowledge regarding the molecular subtypes and genetic landscape of pancreatic cancer and their clinical impact. Then, we discuss some drugs that have been demonstrated to have potential clinical impact when repurposed for PDAC treatment highlighting recently completed and some ongoing clinical trials. Ultimately, we highlight the challenges of repurposing drugs for PDAC treatment and the opportunities for overcoming them.

## 2 Molecular subtypes of PDAC

Several studies have stratified patients into distinct subtypes using cutting-edge techniques such as single-cell analyses (Pompella et al., 2020) (Table 1). For example, Collision et al. classified PDAC into three subtypes, namely, quasi-mesenchymal subtype which is the most aggressive, exocrine-like subtype, and the classical subtype with the best prognosis of all three (Collisson et al., 2011). They employed microarray gene expression for human and cell line models to pinpoint the genetic signature underlying each subtype. Bailey et al. (2016) identified four subtypes of PDAC (pancreatic progenitor, squamous, aberrantly differentiated endocrine exocrine (ADEX), and immunogenic) according to an integrated genomic analysis of 456 pancreatic cancer patients using a combination of whole genome, deep exome sequencing, and transcriptional profile. Another study used non-negative matrix factorisation to perform virtual microdissection of microarray results for PDAC categorization in two tumour-specific groups: classical and basal-like (Moffitt et al., 2015). Similarly, using integrated multi-omics profiling of 150 PDAC specimens two subtypes were identified as basal-like squamous and classical/pancreatic progenitors (Raphael et al., 2017). Most recently Chan-Seng-Yue and colleagues used a combination of whole genome and transcriptome sequencing as well as single-cell sequencing to classify the tumours into Basal-like A and B, hybrid, and classical A and B subtypes (Chan-Seng-Yue et al., 2020).

**TABLE 1:** Impact of the classification of tumour subtypes in the genetic events of PDAC.

Subtypes	Impact of Genomic Mutations
Basal-like	• Complete loss of CDKN2A Chan-Seng-Yue et al. (2020).
	• Elevated frequency of TP53 mutations Raphael et al. (2017).
	• KRAS mutation is stage-dependent: Metastatic basal-like tumours are enriched with KRAS mutants Chan-Seng-Yue et al. (2020).
	• SMAD4 gene alterations: SMAD4 gene is a key player in TGF-β signalling which is elevated in basal-like tumours.
Squamous	• Major KRAS imbalances at the late stage of PDAC Chan-Seng-Yue et al. (2020).
Classical	• Elevated frequency of complete loss of SMAD4 Bailey et al. (2016), Chan-Seng-Yue et al. (2020).
	• GATA6 amplification Collisson et al. (2011).
ADEX	• Upregulation of genes involved in KRAS activation Bailey et al. (2016).
Immunogenic	• Elevated levels of genes associated with B and T immune cell populations Maurer et al. (2019), Zhou et al. (2021).

The classical subtype is characterized by high expression of adhesion-associated and epithelial genes such as GATA binding protein 6 (*GATA6*), *KRAS*, and *SMAD4* in addition to *KRAS* G12V high mutational level (Collisson et al., 2011; Moffitt et al., 2015). Progenitor is similar to the classical subtype and defined by expressing genes associated with early tumour development like steroid hormone biosynthesis, fatty acid oxidation, drug metabolism, and O-linked glycosylation of mucins (Bailey et al., 2016). The squamous subtype is characterized by inflammation, hypoxia, TGF-β signalling, metabolic reprogramming, and activation of the MYC pathway which are linked to poor outcomes (Bailey et al., 2016). The pancreatic squamous basal-like tumours were shown to be associated with mutations in *TP53* that are essential in driving epithelial to mesenchymal transition (EMT) promoting metastasis (Raphael et al., 2017).

## 3 Clinical impact of molecular subtypes

Recent studies have demonstrated that different subtypes can impact on clinical outcomes of pancreatic patients (Dreyer et al., 2022). PDAC patients with the classical subtype were shown to have a better prognosis than those with the quasi-mesenchymal subtype after resection which could be due to the elevated expression of mesenchyme-associated genes and decreased GATA6 levels in the latter (Collisson et al., 2011). Pancreatic cancer development has been associated with the overexpression of GATA6 (Fu et al., 2008). Subtype-associated with long intergenic non-coding RNA (lincRNAs) may be vital in predicting the overall survival rate in PDAC via the subtype-specific selection burden on GATA6 (Glaß et al., 2020). Additionally, GATA6 could be used as a marker of response to chemotherapy because it regulates EMT and tumour dissemination (Martinelli et al., 2017; Deng et al., 2020).

The relationship between KRAS dependence and subtypes was assessed using RNA interference (RNAi) to probe KRAS-mutant human PDAC cell lines and it showed classical subtypes are more dependent on KRAS than quasi-mesenchymal (Collisson et al., 2011). This suggests that KRAS-directed therapy could be vital in the classical PDAC subtype. The response of PDAC to chemotherapy is influenced by their subtypes; quasi-mesenchymal was shown to be more sensitive to gemcitabine while erlotinib was more effective in classical PDAC cell lines (Torres and Grippo, 2018).

ADEX subgroup identification is vital in the later stages of pancreatic development and differentiation via the upregulation of genes that regulate networks, such as NR5A2 involved in KRAS activation (Bailey et al., 2016; von Figura et al., 2014). The progenitor subtype is associated with IPMN and better survival than other subtypes (Bailey et al., 2016) while the squamous subtypes were significantly associated with poorer survival than ADEX and immunogenic subtypes (Hong et al., 2021). Furthermore, the immunogenic subtype is associated with immune cell infiltrates and cellular programs such as antigen presentation, B cell signalling pathway, CD4 and CD8 T-cells (Rooney et al., 2015; Bailey et al., 2016).

## 4 Key genomic aberrations in PDAC

### 4.1 Kirsten rat sarcoma viral oncogene homolog (KRAS)

*KRAS* pathway has been one of the most characterised pathways in PDAC. Genetic approaches have demonstrated that *KRAS* mutations occur in over 95% of PDAC tumours (Dreyer et al., 2017). A high mutational level of the KRAS gene in PDAC has been linked to disease initiation, growth, and progression (Buscail et al., 2020). They are deactivated when they bind to GDP and activated when GTP is attached. Activated *KRAS* initiates *RAS* kinase which in turn genetically dysregulates multiple pathways in PDAC that could serve as potential therapeutic targets. Until recently, *KRAS* has been tagged undruggable because it lacks a binding site for a competitive inhibitor (Gillson et al., 2020). Recently, AMG 510 has been developed as a drug used to maintain a high level of inactive *KRAS* by binding to only reactive mutant *KRAS* ^G12C^ (Canon et al., 2019). Phase I/II clinical trials involving 533 patients with *KRAS* ^G12C^ mutations from various cancers were carried out to investigate the efficacy of AMG510 as monotherapy or in combination with anti-PD-1 immune checkpoint blockers showed that 56% partial response and 46% stable disease (Govindan et al., 2019). About 60% of *KRAS* wild types have altered activation of the RAS-MAPK pathway, hence highlighting the importance of this pathway in therapy development (Martinelli et al., 2017). A drug called Compound 11 can disrupt KRAS interaction with Raf effector, inhibiting the MAPK growth pathway (McCarthy et al., 2019) which could be a potential therapy for PDAC. Another compound, MRTX849 was used in phase I clinical trials in patients with advanced cancers, and results demonstrated a high percentage of partial response (Hallin et al., 2020). Most recently, *KRAS* G12D inhibitor MRTX1133 have been shown to inhibit oncogenic *KRAS* signalling and reduce tumour growth via the inactivation of ERK pathway (Mao et al., 2022; Wang et al., 2022).

### 4.2 Tumour protein 53 (TP53)

*TP53* mutations occur in over 70% of PDAC initiating the activation of KRAS mutation and are usually associated with poor outcomes (Masetti et al., 2018; Liu et al., 2021). They result in the loss of both DNA binding ability and gene transcription activation (Kern et al., 1992). *TP53* is mutated and not deleted in most cancers which is achieved by *p21* gene activation promoting growth arrest (Morton et al., 2010). It also increases the expression of cyclin-dependent kinase inhibitor (CDKN1A) which inhibits cell cycle progression (Cicenas et al., 2017). Class 1 and 2 histones deacetylases (HDAC1/HDAC2) which have been linked to metastasis and treatment resistance in PDAC also promote the expression of P53 in PDAC. Thus, a combined approach targeting the inhibition of HDAC1 and HDAC2 could be vital in the development of therapy (Stojanovic et al., 2017). The depletion of HDAC2 has been proven to induce apoptosis of pancreatic cancer cell lines (Schüler et al., 2010). Circulating *TP53* has been linked to poor survival in PDAC patients treated with FOLFIRINOX (van der Sijde et al., 2021). A Phase II clinical trial (NCT02340117) study of combined targeted P53 gene therapy (SGT-53) with Gemcitabine/Nab-Paclitaxel for the treatment of metastatic pancreatic cancer is currently ongoing (Leung et al., 2021).

### 4.3 Cyclin-dependent kinase 2A (CDKN2A)

*CDKN2A* is a gene located at chromosome 9 which encodes proteins that control cell proliferation and their mutation increases the risk of pancreatic cancer (Hu et al., 2018). Germline *CDKN2A* variants are present in over 3% of pancreatic tumours which demonstrates their importance in carcinogenesis (Kimura et al., 2021). The inactivation of *CDKN2A* is mediated by the methylation of its promoter region. *CDKN2A* encodes two proteins; p14 and p16 which are responsible for cell cycle arrest, DNA repair constraints cyclin-dependent kinase 6 (*CDK6*) and cyclin-dependent kinase 4 (*CDK4*) which triggers the activation blocks G1 to S phase (Knudsen et al., 2016). Alterations of *CDKN2A* induce cyclin-dependent kinase 4 and 6 (CDK4/6) activity and lead to cell proliferation (Kimura et al., 2021). Consequently, inhibition of CDK4/6 could be a potential target in anti-tumour therapy in PDAC patients. Clinical studies targeting *CDK4/6* inhibition in PDAC patients with *CDKN2A* loss or mutation (Clinical trials: NCT02501902, NCT02897375) have been demonstrated to be crucial in PDAC therapy (Al Baghdadi et al., 2019; Hidalgo et al., 2020).

### 4.4 Mothers against decapentaplegic homolog 4 (SMAD4)

*SMAD4* is a tumour suppressor protein also known as DPC4 (deleted in pancreatic cancer 4). *SMAD4* mutations occur in over 50% of PDAC cases promoting invasion, metastasis and poor prognosis via the inhibition of TGF-β signalling pathways by affecting cell arrest, apoptosis, invasion and metastasis (Huang et al., 2020). The correlation between *SMAD4* gene inactivation with survival time suggests a poor prognosis in PDAC (Blackford et al., 2009; Singh et al., 2012). Resistance to PDAC treatment has been associated with *SMAD4* loss limiting the vulnerability of pancreatic cancer cells to complex I inhibition via the promotion of mitophagy (Ezrova et al., 2021). Genomic and transcriptomic profiling analyses demonstrated that classical and progenitor subtypes are enriched with *SMAD4* mutations compared to other subtypes (Chan-Seng-Yue et al., 2020).

## 5 Repurposing drugs in PDAC treatment

**FIGURE 1:**
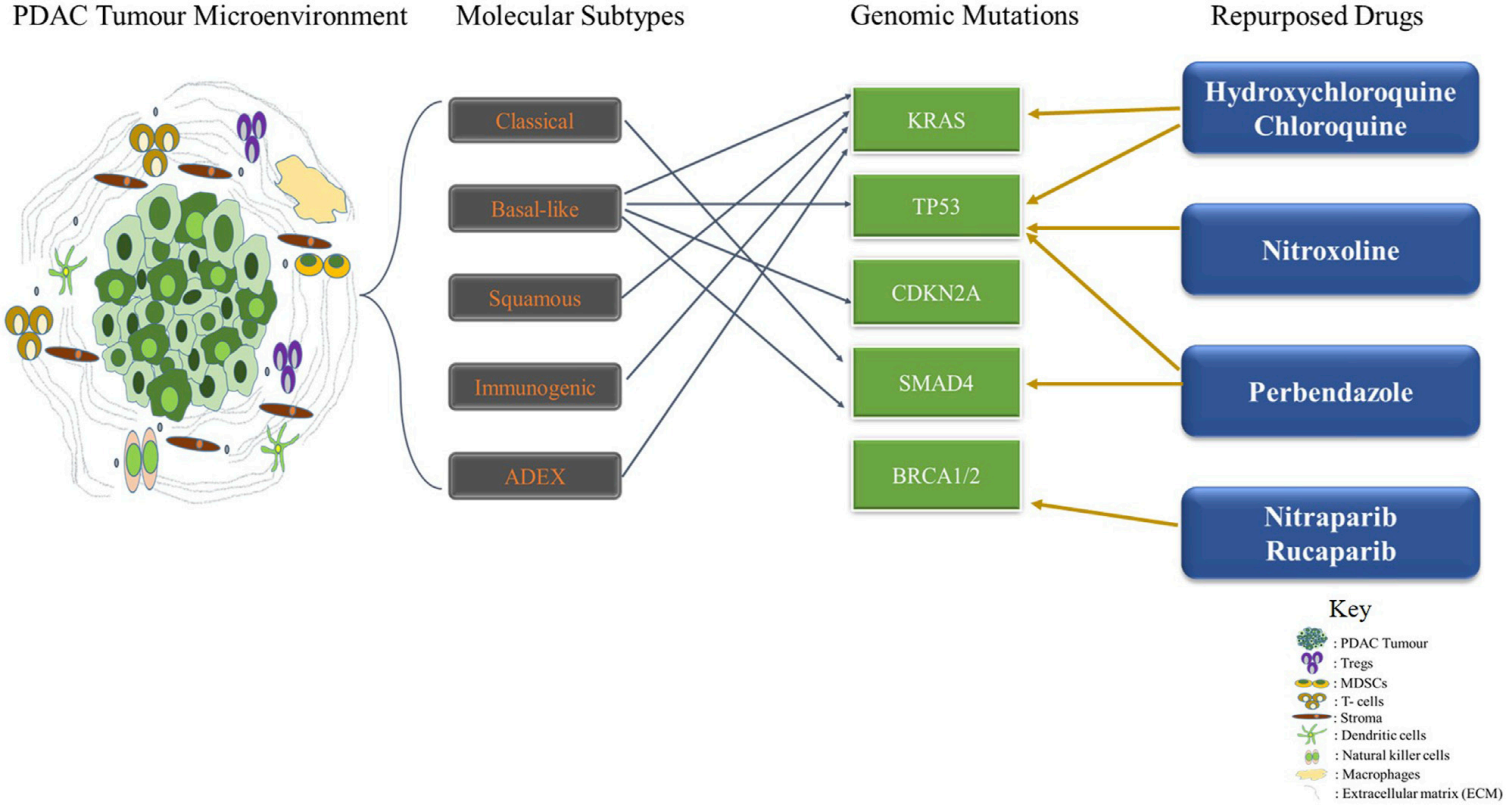
Drug Repurposing Therapy in Pancreatic Ductal Adenocarcinoma Targeting Genomic Alterations and Molecular Subtypes. The Tumor microenvironment of PDAC is complex and heterogeneous. Several molecular subtypes such as classical, basal-like, squamous, immunogenic and ADEX have been identified thus far with diverse molecular perturbations. Repurposed drugs such as hydroxychloroquine, chloroquine, Nitroxoline, Perbendazole, Nitraparib, and Rucaparib are used in targeting genomic mutations in PDAC.

Drug repurposing also known as drug redirection and therapeutic switching involves the process of identifying new therapeutic use for old or existing drugs. It provides a solution to the time-consuming, laborious, expensive, and high-risk process of traditional drug discovery (Pushpakom et al., 2019; Nweke et al., 2021). These drugs can target single or multiple aberrations in targets or pathways which may in turn circumvent resistance (Sarmento-Ribeiro et al., 2019) (Figure 1; Table 2).

**TABLE 2:** Some repurposed drug for PDAC treatment.

Drug	Original Indication	New Indication	Preclinical/Clinical Studies
Cucurmin	Colouring agent in food	Curcumin has been shown to modulate various pathways which are dysregulated in PDAC, they have an anticancer effect either as a single agent or in combination with other chemotherapy drugs Hosseini et al. (2017).	A phase I/II gemcitabine-based chemotherapy in combination with curcumin for treating Pancreatic cancer patients showed that the drug is well tolerable with increased efficacy Kanai et al. (2011).
		Antiproliferative effect by induction of apoptosis and inhibition of both angiogenesis and oxidative stress Bimonte et al. (2016), Nagaraju et al. (2019).	
Genistein	Phytoestrogen is used in the dietary management of skin health Irrera et al. (2017).	Exerts antitumour activity in pancreatic cancer cells via regulation of STAT3, cell cycle arrest, and ROS-mediated apoptosis Bi et al. (2018).	Both *in vivo* and *in vitro* studies showed that a combination of 5- Fluorouracil and genistein have more antitumour effects on human pancreatic cancer cells than when compared with either 5- Fluorouracil or genistein alone Suzuki et al. (2014).
			Phase I clinical trial of AXP107-11, a crystalline component form of genistein in combination with gemcitabine for unresectable pancreatic cancer patients showed that 44% of the patients survived longer than 6 months and 19% were alive after one year Löhr et al. (2016).
Spironolactone	Treatment of hypertension and heart failure Kosmas et al. (2018).	Inhibits DNA repair and acts as a chemosensitizer in combination with DNA-damaging reagents such as cisplatin Gold et al. (2019).	Decreases resistance to Gemcitabine and Osimertinib in pancreatic cancer cell lines Sanomachi et al. (2019).
		Reducing survivin expression which is an anti-apoptotic protein Sanomachi et al. (2019).	
Parbendazole and Mebendazole	Anti-parasitic agents Son et al. (2020).	Promotes Apoptosis, DNA damage, and impairs cell migration Florio et al. (2019).	A phase 2a clinical study on the safety and efficacy of using mebendazole in treating gastrointestinal cancer showed high tolerability of the drugs Mansoori et al. (2021).
		Reduces pancreatic tumour size and inhibits liver metastasis Williamson et al. (2021).	
Berberine	Reduce blood glucose and insulin levels in type 2 Diabetes mellitus patients Cicero and Baggioni (2016).	Inhibits pancreatic cancer cell viability and metastases by regulating citrate metabolism Liu et al. (2020).	Clinical trials (NCT03281096) using Berberine Hydrochloride in Colorectal cancer patients are still ongoing.
		The proliferation of PDAC cells, retarding the development of their cycle in G1 and DNA synthesis inhibition Rauf et al. (2021).	Clinical trials (NCT03333265) Primary chemoprevention of Familial Adenomatous Polyposis with Berberine Hydrochloride.
Niclosamide	Treating tapeworm infections Kadri et al. (2018).	Exerts anticancer effects by inhibiting proliferation Kaushal (2021).	Reduces immune evasion and induces GSK-β mediated β-catenin degradation to promote gemcitabine activity which suppresses pancreatic cancer progression Guo et al. (2022).
		Elevated levels of Niclosamide induce apoptosis of pancreatic cancer cells via the mitochondrial apoptotic pathway Guo et al. (2022).	
Ritonavir	Protease inhibitors for controlling HIV infection Cameron et al. (1998).	Inhibition of E2F and AKT pathways promotes the induction of apoptosis and cell cycle arrest Batchu (2014).	Potential use of ritonavir in combination with chemotherapy in human pancreatic tumour cell lines Batchu (2014).
Itraconazole	Antifungal infections Piérard et al. (2000).	Induced apoptosis via ROS generation Jiang et al. (2019).	Activates apoptosis via inhibition of TGF-β /SMAD2/SMAD3 signaling in pancreatic cancer cell lines Chen et al. (2018).
Disulfiram	Treatment of Alcoholism Chick et al. (1992).	Inhibition of the NF-κB pathway downregulates the stem genes Cong et al. (2017).	Disulfiram synergises with SRC inhibitors to suppress the growth of PDAC cells *in vitro* and *in vivo* Li et al. (2021).
Bazedoxifene	Treatment of osteoporosis Yavropoulou et al. (2019).	Activates IL-6 and IL-11 which mediates inhibition of STAT3 Wu et al. (2016).	Bazedoxifene is a potential new therapeutic option for PDAC treatment that is safe to use and at a low cost Burkhardt et al. (2019).

### 5.1 Aspirin

Notably, drugs such as aspirin, a non-steroidal anti-inflammatory (NSAID) medication have been shown to have anti-neoplastic effects because of their ability to inhibit the prostaglandins precursors, COX-1 and COX-2 enzymes that regulate inflammatory processes (Sleire et al., 2017). Acetylation of aspirin inhibits activation of the transcription factor NF-κB that regulates the expression of genes involved in apoptosis and metastasis (Sleire et al., 2017). Risch and colleagues demonstrated an inverse relationship between the use of Aspirin and the risk of pancreatic cancer in a Chinese cohort (Risch et al., 2017). Zhang’s team demonstrated the anticancer effects of Aspirin on PDAC cell lines. This study recorded the multifunction of Aspirin to alter the expression of reprogramming factors, increase the efficacy of gemcitabine, inhibit tumour growth, and reduce the production of extracellular matrix components such as collagen and fibronectin (Zhang et al., 2015). Aspirin can inhibit neuraminidase-1 (Neu-1) which regulates the activation of toll-like receptors, several receptor tyrosine kinases, and their signalling pathways (Haxho et al., 2016). Targeting Neu-1 by using repurposed drugs could be of potential for inhibiting proliferation and tumorigenesis in PDAC (Qorri et al., 2022). Recent studies showed that a combination of Aspirin, Oseltamivir Phosphate and Gemcitabine could promote the inhibition of survival pathways required for progression in Pancreatic cancer cell lines (Qorri et al., 2020).

### 5.2 Metformin

This is an oral biguanide used to treat diabetes but is associated with decreased overall cancer incidence (Gandini et al., 2014). The anti-neoplastic effect of metformin could be due to the inhibition of mTOR and ROS (Candido et al., 2018). It is also associated with DNA damage and activation of AMPK (Algire et al., 2012). Metformin has been demonstrated to have an antitumour effect on PDAC cells by suppressing hepatic nuclear factor gamma (HNF4G) activity and could be a target for precision treatment (Wang et al., 2021b). A phase 2 trial (NCT01210911) and recent meta-analysis studies have shown the significant role of metformin in improving the overall survival rate in advanced pancreatic cancer patients (Dulskas et al., 2020; Shi et al., 2020). Although many studies have suggested that metformin might improve survival in PDAC (Amin et al., 2016; Cerullo et al., 2016), several are contrary (Kordes et al., 2015; Chaiteerakij et al., 2016; Reni et al., 2016) hence further prospective and clinical trials are essential to confirm these findings.

### 5.3 Vitamins

Vitamin D is a fat-soluble steroid that can be gotten from sun exposure diet and dietary supplements, and are responsible for increasing intestinal absorption of calcium, magnesium, and phosphate. Vitamins D_2_ and D_3_ are the most important compounds in this group. Studies have demonstrated that Vitamin D reduces the risk of pancreatic cancer by regulating cell cycle and differentiation. Although the effect of Vitamin D on the molecular mechanism underlying pancreatic cancer development is not well understood, Vitamin D receptors (VDR) are involved in several metabolic pathways, immune responses, and malignancies (Cannon et al., 2016). Treating PDAC mice with a high dose of calcipotriol, an analogue of Vitamin D has demonstrated that VDR could modulate inflammatory cytokines and growth factors and reduce inflammation and fibrosis (Sherman et al., 2014). Several clinical trials (NCT03472833 and NCT00238199) have been carried out to investigate the use of high-dose Vitamin D/Calcitriol in PDAC and results showed that Vitamin D could be used as standard therapy in pancreatic cancer patients (Ng et al., 2019; Katayama et al., 2020).

Vitamin C is water-soluble vitamin commonly gotten from fruits and vegetables. Ascorbic acid can act as an antioxidant, immunomodulator, and anti-cancer. Hence, Vitamin C derivatives have been repurposed for cancer therapy as shown in several PDAC clinical studies in which they are either used alone or in combination with conventional chemotherapy (Hirschfeld and Bruckner, 2016; Polireddy et al., 2017).

### 5.4 Beta blockers

These are drugs that prevent the stimulation of adrenergic receptors. Beta adrenoreceptors are G-protein coupled receptors that are expressed by pancreatic cancer cells (Weddle et al., 2001) and have been demonstrated to prolong overall survival (Udumyan et al., 2017; Renz et al., 2018). They play an important role in tumour growth, proliferation, and inhibition of apoptosis via protein kinase A (PKA) pathways (Upadhyaya et al., 2020). The effects of beta-blockers on cancer prognosis were reported to be associated with improved overall survival among pancreatic cancer patients (Na et al., 2018). Long-term use of beta-blockers such as Atenolol, Propranolol, and Carvedilol is correlated with decreased risk of pancreatic cancer (Saad et al., 2020). Atenolol, a beta blocker used for treating hypertension (Wadworth et al., 1991) could be potentially repurposed to inhibit pancreatic cancer cell growth via the modulation of NF-κB using functional network analysis (Hermawan et al., 2020). Propanolol is also another example of a beta-blocker used for treating hypertension, tremors, and other cardiovascular disorders (Hardison et al., 2016) which has been demonstrated to be a potential repurposed PDAC therapy drug. Phase II randomised placebo-controlled PROSPER trial is currently ongoing to assess the safety of the administration of propranolol and etodolac in resectable PDAC (Hüttner et al., 2020).

### 5.5 Hydroxychloroquine and chloroquine

These are aminoquinoline compounds that are used to treat malaria (Rebelo et al., 2021). Hydroxychloroquine is also used in treating rheumatoid arthritis and lupus (Rebelo et al., 2021). These drugs have been shown to have anti-cancer effects. Chloroquine inhibits autophagy by enhancing the ability of ERK inhibitors to mediate antitumour activities in KRAS-driven PDAC (Bryant et al., 2019). Autophagy is essential in maintaining homeostasis and neutrophil extracellular traps (NETs) formation whereby damaged organelles and other intracellular components are recycled (Boone et al., 2015). Autophagy has also been shown to be vital for PDAC growth and progressions in tumours with P53 alterations (Yang et al., 2014). Also, chloroquine has been demonstrated to inhibit NETs formation thereby reducing the hypercoagulability of murine PDA cells (Boone et al., 2018). Clinical phase II trials (NCT01494155) using hydroxychloroquine and chemoradiation for resectable PDAC. Patients were given hydroxychloroquine in addition to chemoradiotherapy with photons before surgery while only hydroxychloroquine was administered to them afterwards and results showed that about 26 patients survived over 18 months (Raldow et al., 2020). Several clinical trials are currently ongoing or have been carried out (NCT04386057, NCT01978184, NCT04524702, and NCT01273805) to evaluate the role of chloroquine or hydroxychloroquine in combination with other chemotherapy regimens with some results showing that the combination therapy was well tolerated and could be further explored.

### 5.6 Nitroxoline

Nitroxoline is an antibiotic used in treating bacterial and fungal infections such as urinary tract infections (Shim and Liu, 2014). Nitroxoline has been shown to inhibit angiogenesis and tumour growth by promoting the acetylation of P53 (Zhang et al., 2000) and has shown an anticancer effect on pancreatic, leukaemia, and ovarian cancer (Jiang et al., 2011). More recently, Nitroxoline treatment has been shown to downregulate Na^+^/K^+^ ATPase and increases intracellular ROS which further suppresses cell migration and invasion via the inhibition of the P13K/AKT pathway in AsPC-1 pancreatic cancer cell lines (Veschi et al., 2020). Combination therapy involving Nitroxoline, Nelfinavir, and chemotherapy agent erlotinib has shown great potential in improving the treatment of PDAC, hence future studies and trials could be vital (Veschi et al., 2018).

### 5.7 Perbendazole

Widely, Parbendazole is used in treating parasite infections in animals. This drug has been shown to possess antiproliferative effects because it has been shown to inhibit growth, promote apoptosis, and induced DNA damage response in pancreatic cancer cell lines (Florio et al., 2019). Perbendazole drastically interferes with cell cycle progression by promoting G2/M arrest in two pancreatic cancer cell lines AsPC-1 and Capan-2 cells (Florio et al., 2019). P53 mutant AsPC-1 showed decreased cyclin B1 levels, a key component in the control of cell cycle progression from G2 to M phase after Perbendazole treatment (Vogel et al., 2004).

### 5.8 Niraparib and Rucaparib

These are poly ADP ribose polymerase (PARP) inhibitors used as maintenance treatment for advanced ovarian, fallopian tube, or peritoneal cancer responding to platinum-based chemotherapy (Musella et al., 2018; Akay et al., 2021). They induce cytotoxicity by inhibiting PARP enzymatic activities which stimulate the formation of PARP-DNA complexes resulting in DNA damage, apoptosis, and cell death (Lee, 2021). PARP inhibitors target somatic or germline mutations of DNA repair genes such as BRCA1/2, and PALB2 (Akay et al., 2021). Phase II clinical trial studies are currently ongoing including; (NCT03553004) which treats metastatic PDAC patients after previous chemotherapy (NIRA-PANC) with Niraparib, and (NCT03601923) treats patients with a mutation in a DNA repair gene with Nariparib (Kasi et al., 2019). Furthermore, phase 2 trials of PARP inhibitor Rucaparib in pancreatic cancer patients with somatic BRCA mutations or deleterious germline have been done (NCT02042378) and some are still ongoing (NCT03140670) (Domchek et al., 2016; Binder et al., 2019). Rucaparib provided clinical benefits to advanced PDAC patients with BRCA mutation and may be an option earlier in the treatment course (Domchek et al., 2016).

## 6 Challenges and limitations of drug repurposing in PDAC treatment

Drug repurposing capitalises on matching the established mechanisms of existing drugs with the disease profile. Although, the past years have shown a leap in our understanding of the inception and progression of PDAC, the molecular underpinnings are still being unravelled. Hence matching the right drug with the right molecular subtype becomes a big challenge when the intricacies of the disease are only partially understood. Pancreatic cancer is characterised by its high degree of complexity and intra-tumoural heterogeneity (Hayashi et al., 2021). Different molecular subtypes may coexist within a single tumour and the genetic landscapes vary between patients (Cros et al., 2018). This heterogeneity poses a challenge when attempting to identify drugs that can effectively target multiple subtypes or mutations. Additionally, there is still an insufficient number of clinical trials assessing the efficacy of repurposed drugs against PDAC therefore available evidence is limited in terms of patient populations, dosing regimens, and long-term outcomes. The development of drug resistance is a common challenge in cancer treatment (Kurt Yilmaz and Schiffer, 2021) and repurposed drugs might not have been optimised for long-term efficacy in the context of pancreatic cancer. Resistance mechanisms that arise due to the unique genetic landscape of pancreatic cancer could undermine the initial benefits of repurposed therapies (Dagogo-Jack and Shaw, 2018). Also, repurposing drugs can introduce the risk of unanticipated side effects or adverse reactions that were not previously observed in their original use. Ensuring patient safety and minimising these risks becomes a critical concern in repurposing efforts. Finally, drug repurposing often involves using existing medications that are already under patent protection (Talevi and Bellera, 2020). Navigating the complexities of intellectual property rights and licensing agreements can pose significant barriers to repurposing potentially limiting access to certain drugs.

## 7 Conclusion and future perspectives

With the preponderance of enigmatic drug resistance in cancer treatment, there is an unmet need to delve into a new trajectory of therapeutic strategies. Stratification of PDAC into clinically and genetically associated groups could open a gateway for discovering novel biomarkers. New technologies such as single-cell-RNA-sequencing and single-cell-omics could be explored to provide a more comprehensive classification of PDAC patients in the different subtypes based on their biology, prognosis, therapeutic targets, and pharmacologic response to drugs. Strikingly, drug repurposing has been implemented for cancer research to facilitate the drug design process and cost. Repurposing drugs to target genomic alterations in PDAC subtypes is the future perspective that should be leveraged for personalized medicine. Hence more future clinical studies targeting repurposed drugs in PDAC could be beneficial in identifying effective treatments.

## References

[B1] Akay M. Funingana I. G. Patel G. Mustapha R. Gjafa E. Ng T. (2021). An in-depth review of Niraparib in ovarian cancer: mechanism of action, clinical efficacy and future directions. Oncol. Ther. 9 (2), 347–364. 10.1007/s40487-021-00167-z 34363200 PMC8593085

[B2] Al Baghdadi T. Halabi S. Garrett-Mayer E. Mangat P. K. Ahn E. R. Sahai V. (2019). Palbociclib in patients with pancreatic and biliary cancer with CDKN2A alterations: results from the targeted agent and profiling utilization registry study. JCO Precis. Oncol. 3 (3), 1–8. 10.1200/PO.19.00124 35100714

[B3] Algire C. Moiseeva O. Deschênes-Simard X. Amrein L. Petruccelli L. Birman E. (2012). Metformin reduces endogenous reactive oxygen species and associated DNA damage. Cancer Prev. Res. (Phila). 5 (4), 536–543. 10.1158/1940-6207.CAPR-11-0536 22262811

[B4] Amin S. Mhango G. Lin J. Aronson A. Wisnivesky J. Boffetta P. (2016). Metformin improves survival in patients with pancreatic ductal adenocarcinoma and pre-existing diabetes: A propensity score analysis. Official J. Am. Coll. Gastroenterology 111 (9), 1350–1357. 10.1038/ajg.2016.288 PMC5041138 27430290

[B5] Bailey P. Chang D. K. Nones K. Johns A. L. Patch A. M. Gingras M. C. (2016). Genomic analyses identify molecular subtypes of pancreatic cancer. Nature 531 (7592), 47–52. 10.1038/nature16965 26909576

[B114] Batchu R. B. Gruzdyn O. V. Bryant C. S. Qazi A. M. Kumar S. Chamala S. (2014). TI-ritonavir-mediated induction of apoptosis in pancreatic cancer occurs via the RB/E2F-1 and AKT pathways. Pharmaceuticals 7, 46–57. 10.3390/ph7010046 24451403 PMC3915194

[B115] Bi Y. Min M. Shen W. Liu Y. (2018). Genistein induced anticancer effects on pancreatic cancer cell lines involves mitochondrial apoptosis, G0/G1cell cycle arrest and regulation of STAT3 signalling pathway. Phytomedicine 39, 10–16. 10.1016/j.phymed.2017.12.001 29433670

[B116] Bimonte S. Barbieri A. Leongito M. Piccirillo M. Giudice A. Pivonello C. (2016). Curcumin anticancer studies in pancreatic cancer. Nutrients 8, 433. 10.3390/nu8070433 27438851 PMC4963909

[B6] Binder K. A. R. Mick R. O’Hara M. Teitelbaum U. Karasic T. Schneider C. (2019). Abstract CT234: A phase II, single arm study of maintenance rucaparib in patients with platinum-sensitive advanced pancreatic cancer and a pathogenic germline or somatic mutation in BRCA1, BRCA2 or PALB2. Cancer Res. 79 (13), CT234. 10.1158/1538-7445.am2019-ct234 33970687

[B7] Blackford A. Serrano O. K. Wolfgang C. L. Parmigiani G. Jones S. Zhang X. (2009). SMAD4 gene mutations are associated with poor prognosis in pancreatic cancer. Clin. Cancer Res. 15 (14), 4674–4679. 10.1158/1078-0432.CCR-09-0227 19584151 PMC2819274

[B8] Boone B. A. Orlichenko L. Schapiro N. E. Loughran P. Gianfrate G. C. Ellis J. T. (2015). The receptor for advanced glycation end products (RAGE) enhances autophagy and neutrophil extracellular traps in pancreatic cancer. Cancer Gene Ther. 22 (6), 326–334. 10.1038/cgt.2015.21 25908451 PMC4470814

[B9] Boone B. A. Murthy P. Miller-Ocuin J. Doerfler W. R. Ellis J. T. Liang X. (2018). Chloroquine reduces hypercoagulability in pancreatic cancer through inhibition of neutrophil extracellular traps. BMC Cancer 18 (1), 678. 10.1186/s12885-018-4584-2 29929491 PMC6013899

[B10] Bryant K. L. Stalnecker C. A. Zeitouni D. Klomp J. E. Peng S. Tikunov A. P. (2019). Combination of ERK and autophagy inhibition as a treatment approach for pancreatic cancer. Nat. Med. 25 (4), 628–640. 10.1038/s41591-019-0368-8 30833752 PMC6484853

[B117] Burkhardt C. Bühler L. Tihy M. Morel P. Forni M. (2019). Bazedoxifene as a novel strategy for treatment of pancreatic and gastric adenocarcinoma. Oncotarget 10, 3198–3202. 10.18632/oncotarget.26833 31139333 PMC6516716

[B11] Buscail L. Bournet B. Cordelier P. (2020). Role of oncogenic KRAS in the diagnosis, prognosis and treatment of pancreatic cancer. Nat. Rev. Gastroenterology Hepatology 17 (3), 153–168. 10.1038/s41575-019-0245-4 32005945

[B118] Cameron D. W. Heath-Chiozzi M. Danner S. Cohen C. Kravcik S. Maurath C. (1998). Randomised placebo-controlled trial of ritonavir in advanced HIV-1 disease. Lancet 351, 543–549. 10.1016/S0140-6736(97)04161-5 9492772

[B12] Candido S. Abrams S. L. Steelman L. Lertpiriyapong K. Martelli A. M. Cocco L. (2018). Metformin influences drug sensitivity in pancreatic cancer cells. Adv. Biol. Regul. 68, 13–30. 10.1016/j.jbior.2018.02.002 29482945

[B13] Cannon T. L. Ford J. Hester D. Trump D. L. (2016). The incidental use of high-dose vitamin D3 in pancreatic cancer. Case Rep. Pancreat. Cancer 2 (1), 32–35. 10.1089/crpc.2016.0003 30631812 PMC6319684

[B14] Canon J. Rex K. Saiki A. Y. Mohr C. Cooke K. Bagal D. (2019). The clinical KRAS(G12C) inhibitor AMG 510 drives anti-tumour immunity. Nature 575 (7781), 217–223. 10.1038/s41586-019-1694-1 31666701

[B15] Cerullo M. Gani F. Chen S. Y. Canner J. Pawlik T. M. (2016). Metformin use is associated with improved survival in patients undergoing resection for pancreatic cancer. J. Gastrointest. Surg. 20 (9), 1572–1580. 10.1007/s11605-016-3173-4 27255657

[B16] Chaiteerakij R. Petersen G. M. Bamlet W. R. Chaffee K. G. Zhen D. B. Burch P. A. (2016). Metformin use and survival of patients with pancreatic cancer: A cautionary lesson. J. Clin. Oncol. 34 (16), 1898–1904. 10.1200/JCO.2015.63.3511 27069086 PMC4966342

[B17] Chan-Seng-Yue M. Kim J. C. Wilson G. W. Ng K. Figueroa E. F. O’Kane G. M. (2020). Transcription phenotypes of pancreatic cancer are driven by genomic events during tumor evolution. Nat. Genet. 52 (2), 231–240. 10.1038/s41588-019-0566-9 31932696

[B119] Chen K. Cheng L. Qian W. Jiang Z. Sun L. Zhao Y. (2018). Itraconazole inhibits invasion and migration of pancreatic cancer cells by suppressing TGF-β/SMAD2/3 signaling. Oncol. Rep. 39, 1573–1582. 10.3892/or.2018.6281 29484419

[B146] Chick J. Gough K. Falkowski W. Kershaw P. Hore B. Mehta B. (1992). Disulfiram treatment of alcoholism. Br. J. Psychiatry. 161, 84–89. 10.1192/bjp.161.1.84 1638335

[B18] Cicenas J. Kvederaviciute K. Meskinyte I. Meskinyte-Kausiliene E. Skeberdyte A. (2017). KRAS, TP53, CDKN2A, SMAD4, BRCA1, and BRCA2 mutations in pancreatic cancer. Cancers 9 (5), 42. 10.3390/cancers9050042 28452926 PMC5447952

[B147] Cicero A. F. G. Baggioni A. (2016). Berberine and Its Role in Chronic Disease. Adv. Exp. Med. Biol. 928, 27–45. 10.1007/978-3-319-41334-1_2 27671811

[B19] Collisson E. A. Sadanandam A. Olson P. Gibb W. J. Truitt M. Gu S. (2011). Subtypes of pancreatic ductal adenocarcinoma and their differing responses to therapy. Nat. Med. 17 (4), 500–503. 10.1038/nm.2344 21460848 PMC3755490

[B120] Cong J. Wang Y. Zhang X. Zhang N. Liu L. Soukup K. (2017). A novel chemoradiation targeting stem and nonstem pancreatic cancer cells by repurposing disulfiram. Cancer Lett. 409, 9–19. 10.1016/j.canlet.2017.08.028 28864067

[B20] Cros J. Raffenne J. Couvelard A. Poté N. (2018). Tumor heterogeneity in pancreatic adenocarcinoma. Pathobiology 85 (1–2), 64–71. 10.1159/000477773 28787741

[B21] Dagogo-Jack I. Shaw A. T. (2018). Tumour heterogeneity and resistance to cancer therapies. Nat. Rev. Clin. Oncol. 15 (2), 81–94. 10.1038/nrclinonc.2017.166 29115304

[B22] Deng X. Jiang P. Chen J. Li J. Li D. He Y. (2020). GATA6 promotes epithelial-mesenchymal transition and metastasis through MUC1/β-catenin pathway in cholangiocarcinoma. Cell. Death Dis. 11 (10), 860. 10.1038/s41419-020-03070-z 33060563 PMC7567063

[B23] Domchek S. M. Hendifar A. E. McWilliams R. R. Geva R. Epelbaum R. Biankin A. (2016). Rucapanc: an open-label, phase 2 trial of the PARP inhibitor rucaparib in patients (pts) with pancreatic cancer (PC) and a known deleterious germline or somatic BRCA mutation. JCO 34 (15), 4110. 10.1200/jco.2016.34.15_suppl.4110

[B24] Dreyer S. B. Chang D. K. Bailey P. Biankin A. V. (2017). Pancreatic cancer genomes: implications for clinical management and therapeutic development. Clin. Cancer Res. 23 (7), 1638–1646. 10.1158/1078-0432.CCR-16-2411 28373362

[B25] Dreyer S. B. Upstill-Goddard R. Legrini A. Biankin A. V. Jamieson N. B. Chang D. K. (2022). Genomic and molecular analyses identify molecular subtypes of pancreatic cancer recurrence. Gastroenterology 162 (1), 320–324.e4. 10.1053/j.gastro.2021.09.022 34534536 PMC8721486

[B26] Dulskas A. Patasius A. Linkeviciute-Ulinskiene D. Zabuliene L. Smailyte G. (2020). Cohort study of antihyperglycemic medication and pancreatic cancer patients survival. Int. J. Environ. Res. Public Health 17 (17), 6016. 10.3390/ijerph17176016 32824907 PMC7503289

[B27] Elebo N. Fru P. Omoshoro-Jones J. Patrick Candy G. Nweke E. E. (2020). Role of different immune cells and metabolic pathways in modulating the immune response in pancreatic cancer (Review). Mol. Med. Rep. 22 (6), 4981–4991. 10.3892/mmr.2020.11622 33174057 PMC7646946

[B28] Elebo N. Omoshoro-Jones J. Fru P. N. Devar J. De Wet van Zyl C. Vorster B. C. (2021). Serum metabolomic and lipoprotein profiling of pancreatic ductal adenocarcinoma patients of african ancestry. Metabolites 11 (10), 663. 10.3390/metabo11100663 34677378 PMC8540259

[B29] Ezrova Z. Nahacka Z. Stursa J. Werner L. Vlcak E. Kralova Viziova P. (2021). SMAD4 loss limits the vulnerability of pancreatic cancer cells to complex I inhibition via promotion of mitophagy. Oncogene 40 (14), 2539–2552. 10.1038/s41388-021-01726-4 33686239

[B30] Falasca M. Kim M. Casari I. (2016). Pancreatic cancer: current research and future directions. Biochimica Biophysica Acta (BBA) - Rev. Cancer 1865 (2), 123–132. 10.1016/j.bbcan.2016.01.001 26794394

[B31] Florio R. Veschi S. di Giacomo V. Pagotto S. Carradori S. Verginelli F. (2019). The benzimidazole-based anthelmintic Parbendazole: A repurposed drug candidate that synergizes with gemcitabine in pancreatic cancer. Cancers 11 (12), 2042. 10.3390/cancers11122042 31861153 PMC6966614

[B32] Fu B. Luo M. Lakkur S. Lucito R. Iacobuzio-Donahue C. A. (2008). Frequent genomic copy number gain and overexpression of GATA-6 in pancreatic carcinoma. null 7 (10), 1593–1601. 10.4161/cbt.7.10.6565 18769116

[B33] Gandini S. Puntoni M. Heckman-Stoddard B. M. Dunn B. K. Ford L. DeCensi A. (2014). Metformin and cancer risk and mortality: A systematic review and meta-analysis taking into account biases and confounders. Cancer Prev. Res. 7 (9), 867–885. 10.1158/1940-6207.CAPR-13-0424 PMC4154969 24985407

[B34] Gillson J. Ramaswamy Y. Singh G. Gorfe A. A. Pavlakis N. Samra J. (2020). Small molecule KRAS inhibitors: the future for targeted pancreatic cancer therapy? Cancers 12 (5), 1341. 10.3390/cancers12051341 32456277 PMC7281596

[B35] Glaß M. Dorn A. Hüttelmaier S. Haemmerle M. Gutschner T. (2020). Comprehensive analysis of LincRNAs in classical and basal-like subtypes of pancreatic cancer. Cancers 12 (8), 2077. 10.3390/cancers12082077 32727085 PMC7464731

[B121] Gold A. Eini L. Nissim-Rafinia M. Viner R. Ezer S. Erez K. (2019). Spironolactone inhibits the growth of cancer stem cells by impairing DNA damage response. Oncogene 38, 3103–3118. 10.1038/s41388-018-0654-9 30622338

[B36] Govindan R. Fakih M. G. Price T. J. Falchook G. S. Desai J. Kuo J. C. (2019). Phase I study of AMG 510, a novel molecule targeting KRAS G12C mutant solid tumours. Ann. Oncol. 30, v163–v164. 10.1093/annonc/mdz244.008

[B122] Guo Y. Zhu H. Xiao Y. Guo H. Lin M. Yuan Z. (2022). The anthelmintic drug niclosamide induces GSK-β-mediated β-catenin degradation to potentiate gemcitabine activity, reduce immune evasion ability and suppress pancreatic cancer progression. Cell. Death Dis. 13, 112. 10.1038/s41419-022-04573-7 35115509 PMC8814035

[B37] Gutiérrez M. L. Muñoz-Bellvís L. Orfao A. (2021). Genomic heterogeneity of pancreatic ductal adenocarcinoma and its clinical impact. Cancers (Basel) 13 (17), 4451. 10.3390/cancers13174451 34503261 PMC8430663

[B145] Halbrook C. J. Lyssiotis C. A. Pasca di Magliano M. Maitra A. (2023). Pancreatic cancer: Advances and challenges. Cell 186, 1729–1754. 37059070 10.1016/j.cell.2023.02.014 PMC10182830

[B38] Hallin J. Engstrom L. D. Hargis L. Calinisan A. Aranda R. Briere D. M. (2020). The KRASG12C inhibitor MRTX849 provides insight toward therapeutic susceptibility of KRAS-mutant cancers in mouse models and patients. Cancer Discov. 10 (1), 54–71. 10.1158/2159-8290.CD-19-1167 31658955 PMC6954325

[B39] Hardison S. Wan W. Dodson K. M. (2016). The use of propranolol in the treatment of subglottic hemangiomas: A literature review and meta-analysis. Int. J. Pediatr. Otorhinolaryngology 90, 175–180. 10.1016/j.ijporl.2016.09.012 27729127

[B40] Haxho F. Neufeld R. J. Szewczuk M. R. (2016). Neuraminidase-1: A novel therapeutic target in multistage tumorigenesis. Oncotarget 7 (26), 40860–40881. 10.18632/oncotarget.8396 27029067 PMC5130050

[B41] Hayashi A. Hong J. Iacobuzio-Donahue C. A. (2021). The pancreatic cancer genome revisited. Nat. Rev. Gastroenterology Hepatology 18 (7), 469–481. 10.1038/s41575-021-00463-z 34089011

[B42] Hermawan A. Putri H. Utomo R. Y. (2020). Functional network analysis reveals potential repurposing of β-blocker atenolol for pancreatic cancer therapy. DARU J. Pharm. Sci. 28 (2), 685–699. 10.1007/s40199-020-00375-4 PMC7704874 33098056

[B43] Hidalgo M. Carbonero R. G. Lim K. H. Messersmith W. Garrido-Laguna I. Borazanci E. (2020). Abstract CT135: A phase 1b study of palbociclib + nab-paclitaxel in patients (pts) with metastatic adenocarcinoma of the pancreas (PDAC). Cancer Res. 80 (16), CT135. 10.1158/1538-7445.am2020-ct135 PMC10035387 36970055

[B44] Hirschfeld A. Bruckner H. (2016). An open-label phase II trial of G-FLIP (low doses of gemcitabine, 5-FU, leucovorin, irinotecan and oxaliplatin), followed by G-FLIP-DM (G-FLIP + low doses of docetaxel and mitomycin C), used concurrently with ascorbic acid (AA), in patients with advanced pancreatic cancer. JCO 34 (15), e15745. 10.1200/jco.2016.34.15_suppl.e15745

[B45] Hong J. Y. Cho H. J. Kim S. T. Park Y. S. Shin S. H. Han I. W. (2021). Comprehensive molecular profiling to predict clinical outcomes in pancreatic cancer. Ther. Adv. Med. Oncol. 13, 17588359211038478. 10.1177/17588359211038478 34471425 PMC8404641

[B123] Hosseini M. Hassanian S. M. Mohammadzadeh E. ShahidSales S. Maftouh M. Fayazbakhsh H. (2017). Therapeutic potential of curcumin in treatment of pancreatic cancer: current status and future perspectives. J. Cell. Biochem. 118, 1634–1638. 10.1002/jcb.25897 28106283

[B46] Hu C. Hart S. N. Polley E. C. Gnanaolivu R. Shimelis H. Lee K. Y. (2018). Association between inherited germline mutations in cancer predisposition genes and risk of pancreatic cancer. JAMA 319 (23), 2401–2409. 10.1001/jama.2018.6228 29922827 PMC6092184

[B47] Huang W. Navarro-Serer B. Jeong Y. J. Chianchiano P. Xia L. Luchini C. (2020). Pattern of invasion in human pancreatic cancer organoids is associated with loss of SMAD4 and clinical outcome. Cancer Res. 80 (13), 2804–2817. 10.1158/0008-5472.CAN-19-1523 32376602 PMC7335355

[B48] Hüttner F. J. Rooman I. Bouche G. Knebel P. Hüsing J. Mihaljevic A. L. (2020). Pancreatic resection with perioperative drug repurposing of propranolol and etodolac: trial protocol of the phase-II randomised placebo controlled PROSPER trial. BMJ Open 10 (9), e040406. 10.1136/bmjopen-2020-040406 PMC7528424 32998931

[B124] Irrera N. Pizzino G. D’Anna R. Vaccaro M. Arcoraci V. Squadrito F. (2017). Dietary management of skin health: the role of genistein. Nutrients 9, 622. 10.3390/nu9060622 28629129 PMC5490601

[B49] Jiang H. Taggart J. E. Zhang X. Benbrook D. M. Lind S. E. Ding W. Q. (2011). Nitroxoline (8-hydroxy-5-nitroquinoline) is more a potent anti-cancer agent than clioquinol (5-chloro-7-iodo-8-quinoline). Cancer Lett. 312 (1), 11–17. 10.1016/j.canlet.2011.06.032 21899946 PMC3395224

[B125] Jiang F. Xing H.-S. Chen W.-Y. Du J. Ruan Y. Lin A.-Y. (2019). Itraconazole inhibits proliferation of pancreatic cancer cells through activation of Bak-1. J. Cell. Biochem. 120, 4333–4341. 10.1002/jcb.27719 30260036

[B126] Kadri H. Lambourne O. A. Mehellou Y. (2018). Niclosamide, a drug with many (re)purposes. Chem Med Chem 13, 1088–1091. 10.1002/cmdc.201800100 29603892 PMC7162286

[B127] Kanai M. Yoshimura K. Asada M. Imaizumi A. Suzuki C. Matsumoto S. (2011). A phase I/II study of gemcitabine-based chemotherapy plus curcumin for patients with gemcitabine-resistant pancreatic cancer. Cancer Chemother. Pharmacol. 68, 157–164. 10.1007/s00280-010-1470-2 20859741

[B50] Kasi A. Chalise P. Williamson S. K. Baranda J. C. Sun W. Al-Rajabi R. M. T. (2019). Niraparib in metastatic pancreatic cancer after previous chemotherapy (NIRA-PANC): A phase 2 trial. JCO 37 (15), TPS4168. 10.1200/jco.2019.37.15_suppl.tps4168

[B51] Katayama E. S. Hue J. J. Bajor D. L. Ocuin L. M. Ammori J. B. Hardacre J. (2020). Clinical trials in pancreatic cancer: A comprehensive analysis. JCO 38 (15), e16730. 10.1200/jco.2020.38.15_suppl.e16730 PMC7517959 33014285

[B128] Kaushal J. B. Bhatia R. Kanchan R. K. Raut P. Mallapragada S. Ly Q. P. (2021). Repurposing niclosamide for targeting pancreatic cancer by inhibiting hh/gli non-canonical axis of gsk3β. Cancers 13, 3105. 10.3390/cancers13133105 34206370 PMC8269055

[B52] Kern S. E. Pietenpol J. A. Thiagalingam S. Seymour A. Kinzler K. W. Vogelstein B. (1992). Oncogenic forms of p53 inhibit p53-regulated gene expression. Science 256 (5058), 827–830. 10.1126/science.1589764 1589764

[B53] Kimura H. Klein A. P. Hruban R. H. Roberts N. J. (2021). The role of inherited pathogenic CDKN2A variants in susceptibility to pancreatic cancer. Pancreas 50 (8), 1123–1130. 10.1097/MPA.0000000000001888 34714275 PMC8562885

[B54] Kleeff J. Korc M. Apte M. La Vecchia C. Johnson C. D. Biankin A. V. (2016). Pancreatic cancer. Nat. Rev. Dis. Prim. 2 (1), 16022. 10.1038/nrdp.2016.22 27158978

[B55] Knudsen E. S. O’Reilly E. M. Brody J. R. Witkiewicz A. K. (2016). Genetic diversity of pancreatic ductal adenocarcinoma and opportunities for precision medicine. Gastroenterology 150 (1), 48–63. 10.1053/j.gastro.2015.08.056 26385075 PMC5010785

[B56] Kordes S. Pollak M. N. Zwinderman A. H. Mathôt R. A. Weterman M. J. Beeker A. (2015). Metformin in patients with advanced pancreatic cancer: A double-blind, randomised, placebo-controlled phase 2 trial. Lancet Oncol. 16 (7), 839–847. 10.1016/S1470-2045(15)00027-3 26067687

[B129] Kosmas C. E. Silverio D. Sourlas A. Montan P. D. Guzman E. (2018). Role of spironolactone in the treatment of heart failure with preserved ejection fraction. Ann. Transl. Med. 6, 461. 10.21037/atm.2018.11.16 30603649 PMC6312809

[B57] Kurt Yilmaz N. Schiffer C. A. (2021). Introduction: drug resistance. Chem. Rev. 121 (6), 3235–3237. 10.1021/acs.chemrev.1c00118 33757288 PMC8164520

[B58] Lee A. (2021). Niraparib: A review in first-line maintenance therapy in advanced ovarian cancer. Target. Oncol. 16 (6), 839–845. 10.1007/s11523-021-00841-2 34635996 PMC8613118

[B59] Leung C. P. Barve M. A. Wu M. S. Pirollo K. F. Strauss J. F. Liao W. C. (2021). A phase II trial combining tumor-targeting TP53 gene therapy with gemcitabine/nab-paclitaxel as a second-line treatment for metastatic pancreatic cancer. JCO 39 (15), 4139. 10.1200/jco.2021.39.15_suppl.4139

[B130] Li Z. Xie X. Tan G. Xie F. Liu N. Li W. (2021). Disulfiram synergizes with SRC inhibitors to suppress the growth of pancreatic ductal adenocarcinoma cells *in vitro* and *in vivo* . Biol. Pharm. Bull. 44, 1323–1331. 10.1248/bpb.b21-00353 34471060

[B131] Liu J. Luo X. Guo R. Jing W. Lu H. (2020). Cell metabolomics reveals berberine-inhibited pancreatic cancer cell viability and metastasis by regulating citrate metabolism. J. Proteome Res. 19, 3825–3836. 10.1021/acs.jproteome.0c00394 32692565

[B60] Liu X. Chen B. Chen J. Sun S. (2021). A novel tp53-associated nomogram to predict the overall survival in patients with pancreatic cancer. BMC Cancer 21 (1), 335. 10.1186/s12885-021-08066-2 33789615 PMC8011162

[B132] Löhr J.-M. Karimi M. Omazic B. Kartalis N. Verbeke C. S. Berkenstam A. (2016). A phase I dose escalation trial of AXP107-11, a novel multi-component crystalline form of genistein, in combination with gemcitabine in chemotherapy-naive patients with unresectable pancreatic cancer. Pancreatology 16, 640–645. 10.1016/j.pan.2016.05.002 27234064

[B133] Mansoori S. Fryknäs M. Alvfors C. Loskog A. Larsson R. Nygren P. (2021). A phase 2a clinical study on the safety and efficacy of individualized dosed mebendazole in patients with advanced gastrointestinal cancer. Sci. Rep. 11, 8981. 10.1038/s41598-021-88433-y 33903692 PMC8076239

[B61] Mao Z. Xiao H. Shen P. Yang Y. Xue J. Yang Y. (2022). KRAS(G12D) can be targeted by potent inhibitors via formation of salt bridge. Cell. Discov. 8 (1), 5. 10.1038/s41421-021-00368-w 35075146 PMC8786924

[B62] Martinelli P. Carrillo-de Santa Pau E. Cox T. Sainz B. Dusetti N. Greenhalf W. (2017). GATA6 regulates EMT and tumour dissemination, and is a marker of response to adjuvant chemotherapy in pancreatic cancer. Gut 66 (9), 1665–1676. 10.1136/gutjnl-2015-311256 27325420 PMC5070637

[B63] Masetti M. Acquaviva G. Visani M. Tallini G. Fornelli A. Ragazzi M. (2018). Long-term survivors of pancreatic adenocarcinoma show low rates of genetic alterations in KRAS, TP53 and SMAD4. Cancer Biomarkers 21 (2), 323–334. 10.3233/CBM-170464 29103024 PMC13078277

[B134] Maurer C. Holmstrom S. R. He J. Laise P. Su T. Ahmed A. (2019). Experimental microdissection enables functional harmonisation of pancreatic cancer subtypes. Gut 68, 1034. 10.1136/gutjnl-2018-317706 30658994 PMC6509007

[B64] McCarthy M. J. Pagba C. V. Prakash P. Naji A. K. van der Hoeven D. Liang H. (2019). Discovery of high-affinity noncovalent allosteric KRAS inhibitors that disrupt effector binding. ACS Omega 4 (2), 2921–2930. 10.1021/acsomega.8b03308 30842983 PMC6396121

[B65] Moffitt R. A. Marayati R. Flate E. L. Volmar K. E. Loeza S. G. H. Hoadley K. A. (2015). Virtual microdissection identifies distinct tumor- and stroma-specific subtypes of pancreatic ductal adenocarcinoma. Nat. Genet. 47 (10), 1168–1178. 10.1038/ng.3398 26343385 PMC4912058

[B66] Morton J. P. Timpson P. Karim S. A. Ridgway R. A. Athineos D. Doyle B. (2010). Mutant p53 drives metastasis and overcomes growth arrest/senescence in pancreatic cancer. Proc. Natl. Acad. Sci. U. S. A. 107 (1), 246–251. 10.1073/pnas.0908428107 20018721 PMC2806749

[B67] Musella A. Bardhi E. Marchetti C. Vertechy L. Santangelo G. Sassu C. (2018). Rucaparib: an emerging parp inhibitor for treatment of recurrent ovarian cancer. Cancer Treat. Rev. 66, 7–14. 10.1016/j.ctrv.2018.03.004 29605737

[B68] Na Z. Qiao X. Hao X. Fan L. Xiao Y. Shao Y. (2018). The effects of beta-blocker use on cancer prognosis: A meta-analysis based on 319,006 patients. Onco Targets Ther. 11, 4913–4944. 10.2147/OTT.S167422 30174436 PMC6109661

[B135] Nagaraju G. P. Benton L. Bethi S. R. Shoji M. El-Rayes B. F. (2019). Curcumin analogs: their roles in pancreatic cancer growth and metastasis. Int. J. Cancer 145, 10–19. 10.1002/ijc.31867 30226272

[B69] Ng K. Nimeiri H. S. McCleary N. J. Abrams T. A. Yurgelun M. B. Cleary J. M. (2019). Effect of high-dose vs standard-dose vitamin D3 supplementation on progression-free survival among patients with advanced or metastatic colorectal cancer: the SUNSHINE randomized clinical trial. JAMA 321 (14), 1370–1379. 10.1001/jama.2019.2402 30964527 PMC6459117

[B70] Nsingwane Z. Candy G. Devar J. Omoshoro-Jones J. Smith M. Nweke E. (2020). Immunotherapeutic strategies in pancreatic ductal adenocarcinoma (PDAC): current perspectives and future prospects. Mol. Biol. Rep. 47 (8), 6269–6280. 10.1007/s11033-020-05648-4 32661873

[B71] Nweke E. E. Thimiri Govinda Raj D. B. (2021). “Drug sensitivity and drug repurposing platform for cancer precision medicine,” in Cell biology and translational medicine. Stem cells in development and disease. Editor Turksen K. (Cham: Springer International Publishing), Vol 12, 47–53. (Advances in Experimental Medicine and Biology). 10.1007/5584_2021_622 33629259

[B136] Piérard G. Arrese J. Piérard-Franchimont C. (2000). Itraconazole. null 1, 287–304. 10.1517/14656566.1.2.287 11249550

[B72] Polireddy K. Dong R. Reed G. Yu J. Chen P. Williamson S. (2017). High dose parenteral ascorbate inhibited pancreatic cancer growth and metastasis: mechanisms and a phase I/IIa study. Sci. Rep. 7 (1), 17188. 10.1038/s41598-017-17568-8 29215048 PMC5719364

[B73] Pompella L. Tirino G. Pappalardo A. Caterino M. Ventriglia A. Nacca V. (2020). Pancreatic cancer molecular classifications: from bulk genomics to single cell analysis. Int. J. Mol. Sci. 21 (8), 2814. 10.3390/ijms21082814 32316602 PMC7215357

[B74] Pushpakom S. Iorio F. Eyers P. A. Escott K. J. Hopper S. Wells A. (2019). Drug repurposing: progress, challenges and recommendations. Nat. Rev. Drug Discov. 18 (1), 41–58. 10.1038/nrd.2018.168 30310233

[B75] Qorri B. Harless W. Szewczuk M. R. (2020). Novel molecular mechanism of aspirin and celecoxib targeting mammalian neuraminidase-1 impedes epidermal growth factor receptor signaling Axis and induces apoptosis in pancreatic cancer cells. Drug Des. Devel Ther. 14, 4149–4167. 10.2147/DDDT.S264122 PMC7550724 33116404

[B76] Qorri B. Mokhtari R. B. Harless W. W. Szewczuk M. R. (2022). Next generation of cancer drug repurposing: therapeutic combination of aspirin and Oseltamivir phosphate potentiates gemcitabine to disable key survival pathways critical for pancreatic cancer progression. Cancers (Basel) 14 (6), 1374. 10.3390/cancers14061374 35326525 PMC8946854

[B77] Rahib L. Smith B. D. Aizenberg R. Rosenzweig A. B. Fleshman J. M. Matrisian L. M. (2014). Projecting cancer incidence and deaths to 2030: the unexpected burden of thyroid, liver, and pancreas cancers in the United States. Cancer Res. 74 (11), 2913–2921. 10.1158/0008-5472.CAN-14-0155 24840647

[B78] Raldow A. Lamb J. Hong T. (2020). Proton beam therapy for tumors of the upper abdomen. BJR 93 (1107), 20190226. 10.1259/bjr.20190226 31430202 PMC7066948

[B79] Raphael B. J. Hruban R. H. Aguirre A. J. Moffitt R. A. Yeh J. J. Stewart C. (2017). Integrated genomic characterization of pancreatic ductal adenocarcinoma. Cancer Cell. 32 (2), 185–203.e13. 10.1016/j.ccell.2017.07.007 28810144 PMC5964983

[B137] Rauf A. Abu-Izneid T. Khalil A. A. Imran M. Shah Z. A. Emran T. B. (2021). Berberine as a potential anticancer agent: a comprehensive review. Molecules 26, 7368. 10.3390/molecules26237368 34885950 PMC8658774

[B80] Rebelo R. Polónia B. Santos L. L. Vasconcelos M. H. Xavier C. P. R. (2021). Drug repurposing opportunities in pancreatic ductal adenocarcinoma. Pharmaceuticals 14 (3), 280. 10.3390/ph14030280 33804613 PMC8003696

[B81] Reni M. Dugnani E. Cereda S. Belli C. Balzano G. Nicoletti R. (2016). (Ir)relevance of metformin treatment in patients with metastatic pancreatic cancer: an open-label, randomized phase II trial. Clin. Cancer Res. 22 (5), 1076–1085. 10.1158/1078-0432.CCR-15-1722 26459175

[B82] Renz B. W. Takahashi R. Tanaka T. Macchini M. Hayakawa Y. Dantes Z. (2018). β2 adrenergic-neurotrophin feedforward loop promotes pancreatic cancer. Cancer Cell. 33 (1), 75–90. 10.1016/j.ccell.2017.11.007 29249692 PMC5760435

[B83] Risch H. A. Lu L. Streicher S. A. Wang J. Zhang W. Ni Q. (2017). Aspirin use and reduced risk of pancreatic cancer. Cancer Epidemiol. Biomarkers Prev. 26 (1), 68–74. 10.1158/1055-9965.EPI-16-0508 27999143 PMC5225096

[B84] Rooney M. S. Shukla S. A. Wu C. J. Getz G. Hacohen N. (2015). Molecular and genetic properties of tumors associated with local immune cytolytic activity. Cell. 160 (1), 48–61. 10.1016/j.cell.2014.12.033 25594174 PMC4856474

[B85] Saad A. Goldstein J. Margalit O. Shacham-Shmueli E. Lawrence Y. R. Yang Y. X. (2020). Assessing the effects of beta-blockers on pancreatic cancer risk: A nested case-control study. Pharmacoepidemiol. Drug Saf. 29 (5), 599–604. 10.1002/pds.4993 32196836

[B138] Sanomachi T. Suzuki S. Togashi K. Sugai A. Seino S. Okada M. (2019). Spironolactone, a classic potassium-sparing diuretic, reduces survivin expression and chemosensitizes cancer cells to non-dna-damaging anticancer drugs. Cancers 11, 1550. 10.3390/cancers11101550 31614999 PMC6826935

[B86] Sarmento-Ribeiro A. B. Scorilas A. Gonçalves A. C. Efferth T. Trougakos I. P. (2019). The emergence of drug resistance to targeted cancer therapies: clinical evidence. Drug Resist. Updat. 47, 100646. 10.1016/j.drup.2019.100646 31733611

[B87] Schüler S. Fritsche P. Diersch S. Arlt A. Schmid R. M. Saur D. (2010). HDAC2 attenuates TRAIL-induced apoptosis of pancreatic cancer cells. Mol. Cancer 9 (1), 80. 10.1186/1476-4598-9-80 20398369 PMC2867820

[B88] Sherman M. H. Yu R. T. Engle D. D. Ding N. Atkins A. R. Tiriac H. (2014). Vitamin D receptor-mediated stromal reprogramming suppresses pancreatitis and enhances pancreatic cancer therapy. Cell. 159 (1), 80–93. 10.1016/j.cell.2014.08.007 25259922 PMC4177038

[B89] Shi Y. Q. Zhou X. C. Du P. Yin M. Y. Xu L. Chen W. J. (2020). Relationships are between metformin use and survival in pancreatic cancer patients concurrent with diabetes: A systematic review and meta-analysis. Med. Baltim. 99 (37), e21687. 10.1097/MD.0000000000021687 PMC7489714 32925714

[B90] Shim J. S. Liu J. O. (2014). Recent advances in drug repositioning for the discovery of new anticancer drugs. Int. J. Biol. Sci. 10 (7), 654–663. 10.7150/ijbs.9224 25013375 PMC4081601

[B91] Siegel R. L. Miller K. D. Fuchs H. E. Jemal A. (2022). Cancer statistics, 2022. A Cancer J. Clin. 72 (1), 7–33. 10.3322/caac.21708 35020204

[B92] Singh P. Srinivasan R. Wig J. D. (2012). SMAD4 genetic alterations predict a worse prognosis in patients with pancreatic ductal adenocarcinoma. Pancreas 41 (4), 541–546. 10.1097/MPA.0b013e318247d6af 22504380

[B93] Sleire L. Førde H. E. Netland I. A. Leiss L. Skeie B. S. Enger P. Ø. (2017). Drug repurposing in cancer. Pharmacol. Res. 124, 74–91. 10.1016/j.phrs.2017.07.013 28712971

[B139] Son D.-S. Lee E.-S. Adunyah S. E. (2020). The antitumor potentials of benzimidazole anthelmintics as repurposing drugs. Immune Netw. 20, e29. 10.4110/in.2020.20.e29 32895616 PMC7458798

[B94] Statistics South Africa (2023). Cancer in South Africa, 2008 – 2019 (report No. 03-08-00). Statistics South Africa.

[B95] Stojanovic N. Hassan Z. Wirth M. Wenzel P. Beyer M. Schäfer C. (2017). HDAC1 and HDAC2 integrate the expression of p53 mutants in pancreatic cancer. Oncogene 36 (13), 1804–1815. 10.1038/onc.2016.344 27721407

[B140] Suzuki R. Kang Y. Li X. Roife D. Zhang R. Fleming J. B. (2014). Genistein potentiates the antitumor effect of 5-fluorouracil by inducing apoptosis and autophagy in human pancreatic cancer cells. Anticancer Res. 34, 4685. 25202045 PMC4240628

[B96] Talevi A. Bellera C. L. (2020). Challenges and opportunities with drug repurposing: finding strategies to find alternative uses of therapeutics. Expert Opin. Drug Discov. 15 (4), 397–401. 10.1080/17460441.2020.1704729 31847616

[B97] Torres C. Grippo P. J. (2018). Pancreatic cancer subtypes: A roadmap for precision medicine. Ann. Med. 50 (4), 277–287. 10.1080/07853890.2018.1453168 29537309 PMC6151873

[B98] Udumyan R. Montgomery S. Fang F. Almroth H. Valdimarsdottir U. Ekbom A. (2017). Beta-blocker drug use and survival among patients with pancreatic adenocarcinoma. Cancer Res. 77 (13), 3700–3707. 10.1158/0008-5472.CAN-17-0108 28473530

[B99] Upadhyaya V. D. Douedi S. Garcia B. Gonzalez J. Udongwo N. Wei J. (2020). Mechanism and effect of beta-blockers on pancreatic adenocarcinoma: A literature review. J. Clin. Med. Res. 12 (12), 753–757. 10.14740/jocmr4387 33447308 PMC7781287

[B100] van der Sijde F. Azmani Z. Besselink M. G. Bonsing B. A. de Groot J. W. B. Groot Koerkamp B. (2021). Circulating TP53 mutations are associated with early tumor progression and poor survival in pancreatic cancer patients treated with FOLFIRINOX. Ther. Adv. Med. Oncol. 13, 17588359211033704. 10.1177/17588359211033704 34422118 PMC8377319

[B101] Veschi S. De Lellis L. Florio R. Lanuti P. Massucci A. Tinari N. (2018). Effects of repurposed drug candidates nitroxoline and nelfinavir as single agents or in combination with erlotinib in pancreatic cancer cells. J. Exp. Clin. Cancer Res. 37 (1), 236. 10.1186/s13046-018-0904-2 30241558 PMC6151049

[B102] Veschi S. Ronci M. Lanuti P. De Lellis L. Florio R. Bologna G. (2020). Integrative proteomic and functional analyses provide novel insights into the action of the repurposed drug candidate nitroxoline in AsPC-1 cells. Sci. Rep. 10 (1), 2574. 10.1038/s41598-020-59492-4 32054977 PMC7018951

[B103] Vogel C. Kienitz A. Hofmann I. Müller R. Bastians H. (2004). Crosstalk of the mitotic spindle assembly checkpoint with p53 to prevent polyploidy. Oncogene 23 (41), 6845–6853. 10.1038/sj.onc.1207860 15286707

[B104] von Figura G. Morris J. P. Wright C. V. E. Hebrok M. (2014). Nr5a2 maintains acinar cell differentiation and constrains oncogenic Kras-mediated pancreatic neoplastic initiation. Gut 63 (4), 656–664. 10.1136/gutjnl-2012-304287 23645620 PMC3883808

[B105] Wadworth A. N. Murdoch D. Brogden R. N. (1991). Atenolol. A reappraisal of its pharmacological properties and therapeutic use in cardiovascular disorders. Atenolol. Drugs. 42 (3), 468–510. 10.2165/00003495-199142030-00007 1720383

[B106] Wang S. Zheng Y. Yang F. Zhu L. Zhu X. Q. Wang Z. F. (2021a). The molecular biology of pancreatic adenocarcinoma: translational challenges and clinical perspectives. Signal Transduct. Target. Ther. 6 (1), 249. 10.1038/s41392-021-00659-4 34219130 PMC8255319

[B107] Wang C. Zhang T. Liao Q. Dai M. Guo J. Yang X. (2021b). Metformin inhibits pancreatic cancer metastasis caused by SMAD4 deficiency and consequent HNF4G upregulation. Protein and Cell. 12 (2), 128–144. 10.1007/s13238-020-00760-4 32737864 PMC7862466

[B108] Wang X. Allen S. Blake J. F. Bowcut V. Briere D. M. Calinisan A. (2022). Identification of MRTX1133, a noncovalent, potent, and selective KRASG12D inhibitor. J. Med. Chem. 65 (4), 3123–3133. 10.1021/acs.jmedchem.1c01688 34889605

[B109] Weddle D. L. Tithoff P. Williams M. Schuller H. M. (2001). Beta-adrenergic growth regulation of human cancer cell lines derived from pancreatic ductal carcinomas. Carcinogenesis 22 (3), 473–479. 10.1093/carcin/22.3.473 11238189

[B141] Williamson T. Abreu M. C. Trembath D. G. Brayton C. Kang B. Mendes T. B. (2021). Mebendazole disrupts stromal desmoplasia and tumorigenesis in two models of pancreatic cancer. Oncotarget 12, 1326–1338. 10.18632/oncotarget.28014 34262644 PMC8274724

[B142] Wu X. Cao Y. Xiao H. Li C. Lin J. (2016). Bazedoxifene as a novel GP130 inhibitor for pancreatic cancer therapy. Mol. Cancer Ther. 15, 2609–2619. 10.1158/1535-7163.MCT-15-0921 27535971 PMC5310670

[B110] Yang A. Rajeshkumar N. V. Wang X. Yabuuchi S. Alexander B. M. Chu G. C. (2014). Autophagy is critical for pancreatic tumor growth and progression in tumors with p53 alterations. Cancer Discov. 4 (8), 905–913. 10.1158/2159-8290.CD-14-0362 24875860 PMC4125497

[B143] Yavropoulou M. P. Makras P. Anastasilakis A. D. (2019). Bazedoxifene for the treatment of osteoporosis. null 20, 1201–1210. 10.1080/14656566.2019.1615882 31091133

[B111] Zhang Y. Griffith E. Sage J. Jacks T. Liu J. (2000). Cell cycle inhibition by the anti-angiogenic agent TNP-470 is mediated by p53 and p21WAF1/CIP1. Proc. Natl. Acad. Sci. U. S. A. 97 (12), 6427–6432. 10.1073/pnas.97.12.6427 10841547 PMC18619

[B112] Zhang Y. Liu L. Fan P. Bauer N. Gladkich J. Ryschich E. (2015). Aspirin counteracts cancer stem cell features, desmoplasia and gemcitabine resistance in pancreatic cancer. Oncotarget 6 (12), 9999–10015. 10.18632/oncotarget.3171 25846752 PMC4496413

[B144] Zhou X. Hu K. Bailey P. Springfeld C. Roth S. Kurilov R. (2021). Clinical impact of molecular subtyping of pancreatic cancer. Front. Cell. Dev. Biol. 9, 743908. 10.3389/fcell.2021.743908 34805152 PMC8603393

